# Pamphlets Received

**Published:** 1882-11-20

**Authors:** 


### *PAMPHLETS RECEIVED.*

Life of John M. Briggs, of Bowling Green, Kentucky. By W. K. Bowling, M. D.

The place where born, and the time when,
Have much to do in making men.

The early diagnosis of Chronic Bright's Disease. By T. A. McBride, M. D., of New York.

The application of Pressure in diseases of the Uterus, Ovaries and Peri-Uterine structures. By V. H. Talliaferro, M. D., Atlanta, Georgia, Professor of Obstetrics and Diseases of Women and Children in the Atlanta Medical College.

Abortive treatment of Mammary Abscesses and the cure of Fissured Nipples by means of a new and effectual compress. By George H Noble, M. D., Atlanta, Georgia.

Some observations on the Therapeutic Use of Alcohol. By Alfred K. Hills, M. D., of New York.

Treatment of Consumption, indicated by the discoveries of Koch and others of its Parasitic origin. By M. L. James, M. D., Professor of Materia Medica and Therapeutics in the Medical College of Virginia.

The antiseptic treatment of wounds after operations and injuries. By W. T. Briggs, M. D., Professor of Surgery, Medical Departments of University of Nashville and Vanderbilt University.

Gunshot wound of the abdomen. Fecal fistula—spontaneous closure. Recovery, with remarks on treatment, including a further consideration of the action and applications of quinine. By A. Sibley Campbell, M. D., Augusta, Georgia, Secretary of the Medical Association of Georgia; member of the American Medical Association, etc. Reprinted from the Transactions of the Medical Association of Georgia—Thirty-second annual session, 1881.

Stricture of the Rectum, treated by electrolysis. By Robert Newman, M. D., of New York.

Gonorrhœal Ophthalmia, its Complications and Results ; Iridectomy for Artificial pupil. A clinical lecture at Michigan College Hospital, by C. J. Lundy, M. D., Professor of diseases of the eye, ear and throat.

